# Enhanced Cell Proliferation and Maturation Using Carboxylated Bacterial Nanocellulose Scaffolds for 3D Cell Culture

**DOI:** 10.1021/acsami.4c22475

**Published:** 2025-03-05

**Authors:** Elizabeth Mavil-Guerrero, José Manuel Romo-Herrera, Priscila Quiñonez-Angulo, Francisco J. Flores-Ruiz, Edén Morales-Narváez, J. Félix Armando Soltero, Josué D. Mota-Morales, Karla Juarez-Moreno

**Affiliations:** † Centro de Física Aplicada y Tecnología Avanzada (CFATA), Universidad Nacional Autónoma de México (UNAM), Querétaro 76230, México; ‡ Centro de Nanociencias y Nanotecnología, Universidad Nacional Autónoma de México, Ensenada, Baja California 22800, México; § SECIHTI−Instituto de Física, Benemérita Universidad Autónoma de Puebla, Ciudad Universitaria, Edif. IF-1, Puebla 72570, México; ∥ Centro Universitario de Ciencias Exactas e Ingenierías, Universidad de Guadalajara, Guadalajara, Jalisco 44430, México

**Keywords:** carboxylated bacterial nanocellulose, DES, cell maturation, 3D scaffold, cell culture, 3T3-L1 cells, nanotoxicology

## Abstract

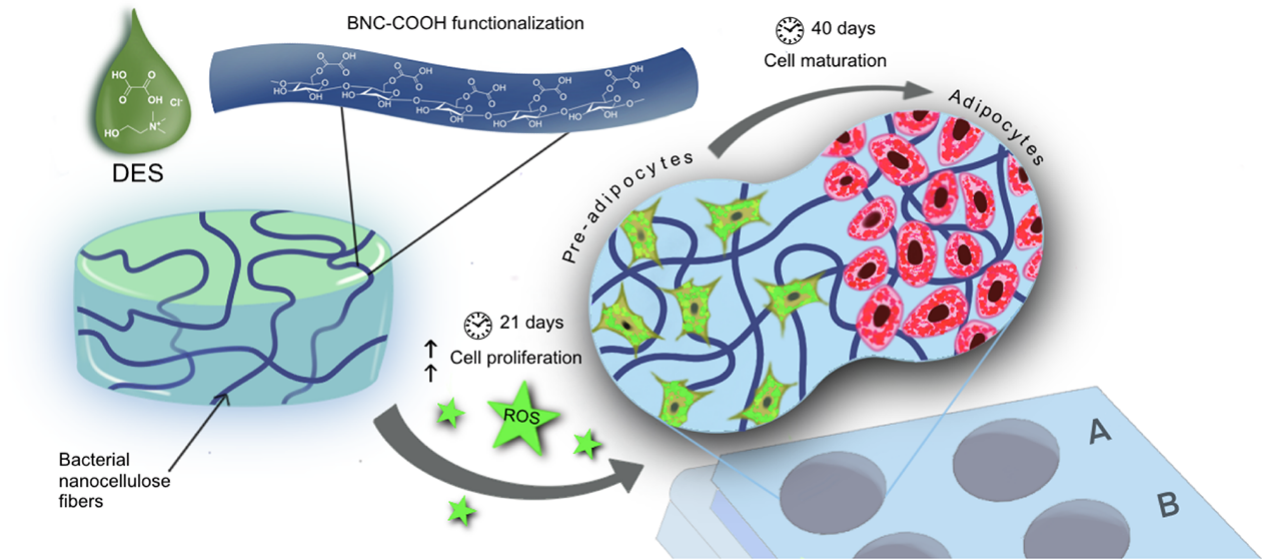

Developing scaffolds for three-dimensional (3D) cell culture and tissue regeneration with biopolymers requires the creation of an optimal nanobiointerface. This interface must possess suitable surface chemistry, biomechanical properties, and fibrillar morphology across nano- to microscale levels to support cell attachment and growth, enabling a biomimetic arrangement. In this study, we developed a hydrogel scaffold made from bacterial nanocellulose (BNC) functionalized with carboxylic acid groups (BNC–COOH) through a reactive deep eutectic solvent (DES), offering a sustainable approach. The surface properties and fibrillar structure of BNC–COOH facilitated the formation of hydrogels with significantly enhanced water uptake (1.4-fold) and adhesion force (2.3-fold) compared to BNC. These hydrogels also demonstrated tissue-like rheological properties in both water with *G*′ exceeding *G*″, suggesting predominantly elastic (solid-like) characteristics and viscosities in the range of 8–15 Pa·s. The BNC–COOH hydrogel scaffold demonstrated excellent biocompatibility, supporting significant cell growth and anchorage for the 3D growth of mammalian cells and enhancing preadipocyte growth by up to 7.3 times. Furthermore, the BNC–COOH hydrogel facilitates the maturation of 3T3-L1 preadipocytes into mature adipocytes, inducing typical morphology changes, such as decreased filopodia extensions, rounded cell shape, and lipid droplet accumulation without any additional chemical induction stimulus. Therefore, we demonstrated that a reactive DES composed of oxalic acid and choline chloride represents a mild reaction medium and a suitable approach for designing biocompatible 3D hydrogel scaffolds with improved physicochemical properties and biological activities for 3D cell culture.

## Introduction

Bacterial nanocellulose (BNC) is being recognized as a highly promising and versatile biobased material that has found diverse applications in areas like energy, environment, food and pharmaceutical sciences, and biomedicine, among others. Currently, BNC applications in biomedicine and hydrogel design have garnered significant attention due to their intrinsic attributes, which include^1^ excellent mechanical properties and biodegradability.^2^ Arranged as a tridimensional network of nanofibrils, BNC exhibits high porosity, shareability, and tissue-like properties, potentially impacting the development of blood-contacting biomedical materials, such as artificial vascular grafts^3,4^ and drug delivery systems.^5^

Compared with other methods for extracting fibers and fibrils from lignocellulosic biomass, the production process for BNC is considerably more straightforward. BNC typically exhibits higher crystallinity, with nanofibril diameters ranging from 20 to 80 nm.^6^ A significant advantage of BNC is its high purity when obtained by biotechnological approaches that involve acetic acid bacteria, as it does not contain lignin or hemicellulose, unlike plant-based nanocellulose. This makes BNC particularly well-suited for designing 3D scaffolds.^6,7^ BNC consists of d-glucose monomeric units linked by glycosidic bonds to form chains arranged in fibrils. The abundance of hydroxyl groups in the chemical structure of BNC enables its cross-linking and functionalization with various peptides, proteins, polysaccharides, and functional groups, improving its chemical and physical properties.^4^ Furthermore, BNC bundles feature networks that resemble the extracellular matrix (ECM) and possess mechanical properties comparable to collagen found in native tissue ECM.^1^

The 3T3-L1 mouse fibroblast cell line is one of the most clearly defined *in vitro* cell culture models for adipocytes, undergoing a transformation from preadipocytes into adipocyte-like cells under specific conditions typically induced by a hormonal cocktail.^8^ This model is crucial for studying adipocyte biology and plays a pivotal role in advancing our understanding of adipogenesis, lipid metabolism, and the effects of hormones and xenobiotics in adipose tissue.^9^ Initial exposure of 3T3-L1 cells to differentiation media triggers the upregulation of adipogenic genes, leading to increased glucose uptake and triglyceride synthesis.^8,10^ It has been documented that 3T3-L1 cells show clear signs of lipid accumulation after the initial exposure to the differentiation medium, and this process may vary between 4 and 7 days.^11^

The 3T3-L1 cells, derived from Swiss 3T3 cells and initially identified by Green et al.,^12^ have been chosen for their capability to accumulate lipids, a key feature for studying adipocyte function. Maturing these preadipocyte cells into full adipocytes requires various agents that promote differentiation.^13^ It has been documented that these cells exhibit a lipid profile characterized by the accumulation of odd-chain-length unbranched fatty acids across all major lipid categories, a process that can be attributed to the α-oxidation of fatty acids in peroxisomes. As these preadipocytes differentiate into adipocytes, there is a notable increase in the concentration of these odd-chain fatty acids.^14^

Several investigations have proposed different BNC-based platforms for cell culture. For instance, Osorio et al.^15^ evaluated the short- and long-term *in vivo* implantation responses of 3D and two-dimensional (2D) porous BNC biomaterials, as well as their *ex vivo* hemocompatibility, including hemolysis and clotting time. They found that biomaterials with porosities of around 60 μm promoted superior fibrotic tissue distribution, high cell migration, and excellent collagen and elastin deposition within the BNC hydrogel. Additionally, Vielreicher et al.^16^ studied the efficiency and quality of collagen-I formation, considering factors such as cell type, medium composition (serum, ascorbic acid), and differences in cell architecture between 2D and 3D cultures. However, most studies have mainly focused on collagen production and have not explored the effect of surface functionalization of BNCs to enhance cell anchorage and biocompatibility.^7^

Deep eutectic solvents (DESs) are an emerging class of designer solvents that have garnered significant attention for their effectiveness in processing and valorizing lignocellulosic materials under sustainable protocols^18,19^ DESs are composed of mixtures of hydrogen bond donors (HBDs) and hydrogen bond acceptors (HBAs), which exhibit enthalpy-driven negative deviations from thermodynamic ideality. In contrast, the melting point depression observed in eutectic solvents results from ideal liquid-phase behavior.^20^ A typical HBA is the quaternary ammonium salt choline chloride (ChCl), while common HBDs encompass a wide range of compounds, including carboxylic acids, polyols, and amides.^21^ Notably, the combination of ChCl and oxalic acid (OA) is widely acknowledged as a DES, recognized for its good biodegradability, accessibility, and low toxicity.^22^ These properties make it particularly suitable for extracting and valorizing nanocellulose and designing materials for biological applications.^19^

This study aimed to design a carboxylated BNC hydrogel (BNC–COOH) for 3D cell culture, using a nonaqueous and sustainable approach at 60 °C of functionalization temperature. To this end, a DES composed of oxalic acid and choline chloride was employed as a reactive solvent for BNC treatment, which introduced carboxylic acid moieties onto the BNC surface to enhance cell anchorage and overall biocompatibility. The mixture of ChCl and OA offers a sustainable process compared with conventional methods that rely on harsh chemicals like TEMPO or strong acids. Moreover, cellulose produced by bacterial genera such as *Gluconacetobacter* is inherently of high purity, eliminating the need for lignin and hemicellulose removal, a step typically required for plant-derived cellulose.^23^

The BNC–COOH hydrogel (ca. 0.1 wt % BNC–COOH) produced in this study was employed as a 3D scaffold for fibroblast cells (3T3-L1). We found that BNC–COOH scaffolds enhanced cell proliferation and induced reactive oxygen species (ROS) production due to higher cellular metabolic activity. Moreover, the 3D architectural arrangement of cells within the BNC–COOH hydrogel scaffold promotes the differentiation of preadipocyte fibroblast cells into adipocytes (3T3-L1) without external chemical stimuli at 40 days. This highlights the key role of surface chemistry in cellulosic forming 3D hydrogels, which can direct the fate of the cell cultured on it. More sustainable biomaterials preparation also represents a step forward in developing BNC hydrogels with prospective applications in tissue engineering and wound healing.

## Results and Discussion

### Morphological and Structure Characterization of Functionalized Bacterial Nanocellulose

DESs offer an environmentally friendly alternative to traditional and harsh solvents for the chemical modification of cellulose. In this study, we extended the results on non-lignocellulosic materials valorization, where oxalic acid and choline chloride-based DES served as an effective reaction medium for the esterification of bacterial nanocellulose fibers’ surface.^18,24^

**Figure 1:**
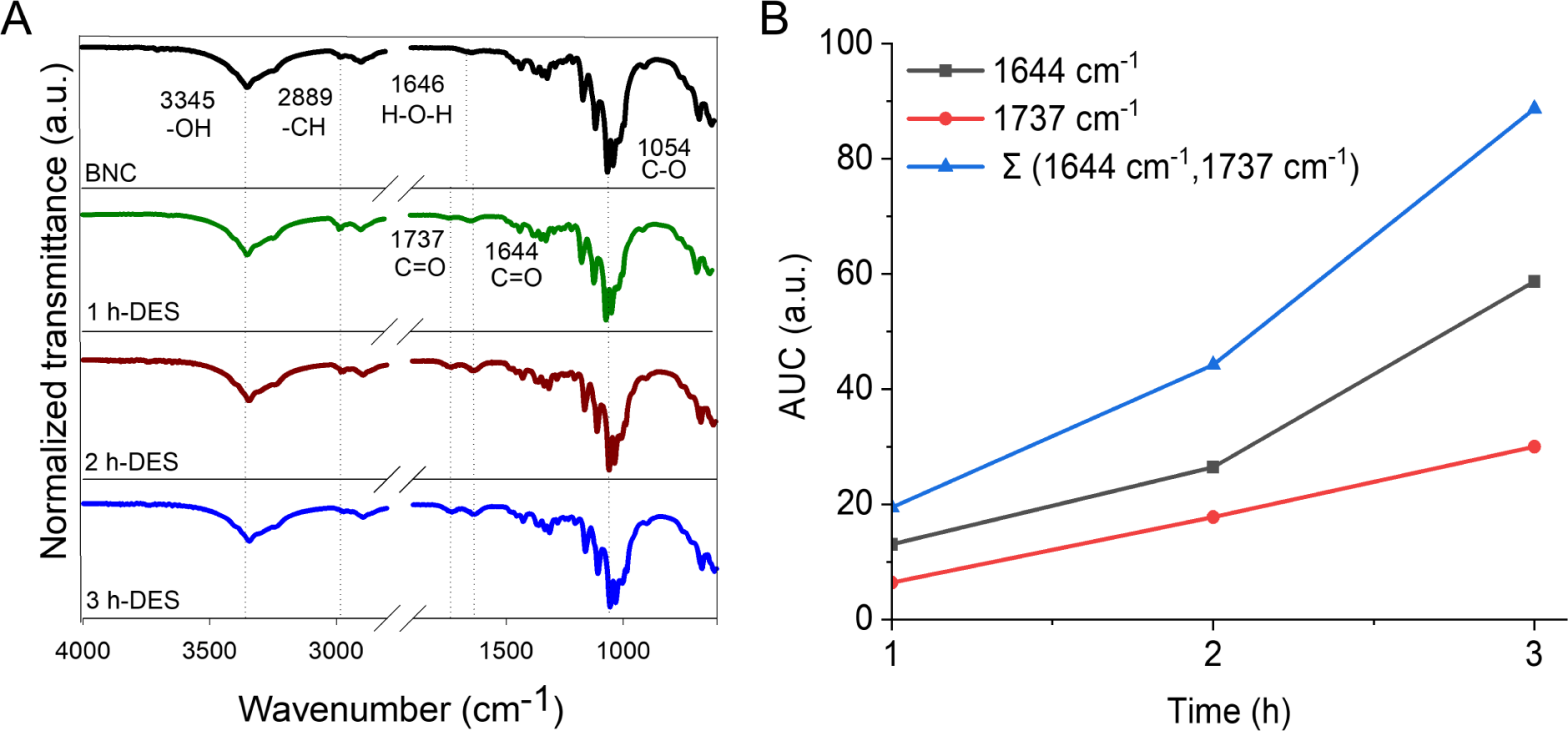
ATR-FTIR spectra of BNC and functionalized BNC–COOH scaffolds are obtained by DES esterification. The reaction was carried out for 1, 2, and 3 h to determine an appropriate functionalization time for the preparation of the scaffold. (A) ATR-FTIR spectra of functionalization of BNC by oxalic acid and choline chloride-based DES at different times. (B) Correlation of –COOH group concentration with the area under the curve (AUC) for the bands at 1644 and 1737 cm^–1^.

To explore the optimal time that yielded the maximum carboxylic acid content in BNC, the treatment was carried out at different times, at a fixed BNC/DES mass ratio of 1:5.^25^Figure 1A shows the characteristic bands of BNC recorded by ATR*-*FTIR after extensively washing out the DES and subsequent drying. The typical band due to −OH stretching is found at 3345 cm^–1^, while the asymmetrical CH_2_ stretching is visible at 2899 cm^–1^. Also, the band at 1054 cm^–1^ resulted from the combination of CH_2_ deformation and C–O–C and C–OH stretching. The band at 1646 cm^–1^ is attributed to the −OH bending of residual water in the BNC.^26−28^Figure 1A shows significant differences in the spectrum of the resulting BNC after the DES treatment. For instance, the bands at 1644 and 1737 cm^–1^ emerged and intensified as the functionalization time increased from 1 to 3 h. These bands correspond to the −C=O and −COOH groups stretching,^18,24^ respectively.

As a first approach to quantify the degree of functionalization, the area under the curve of the bands at 1644 and 1737 cm^–1^ in BNC–COOH at different times was correlated with carboxylic acid moieties’ abundance. The intensity of carbonyl bands increases with longer functionalization times, confirming the modification of the BNC fibrils, likely occurring at the C-6 in the pyranose ring of cellulose using oxalic acid and choline chloride-based DES.^29,30^ The optimal concentration of −COOH groups was achieved after functionalization for 3 h, as indicated by the correlation of the area under the curve of carboxyl bands (Figure 1B). Extended functionalization times resulted in fragile BNC hydrogels, unsuitable as scaffolds for cell culture (Figures S1 and S2 and Movie S1).

**Figure 2:**
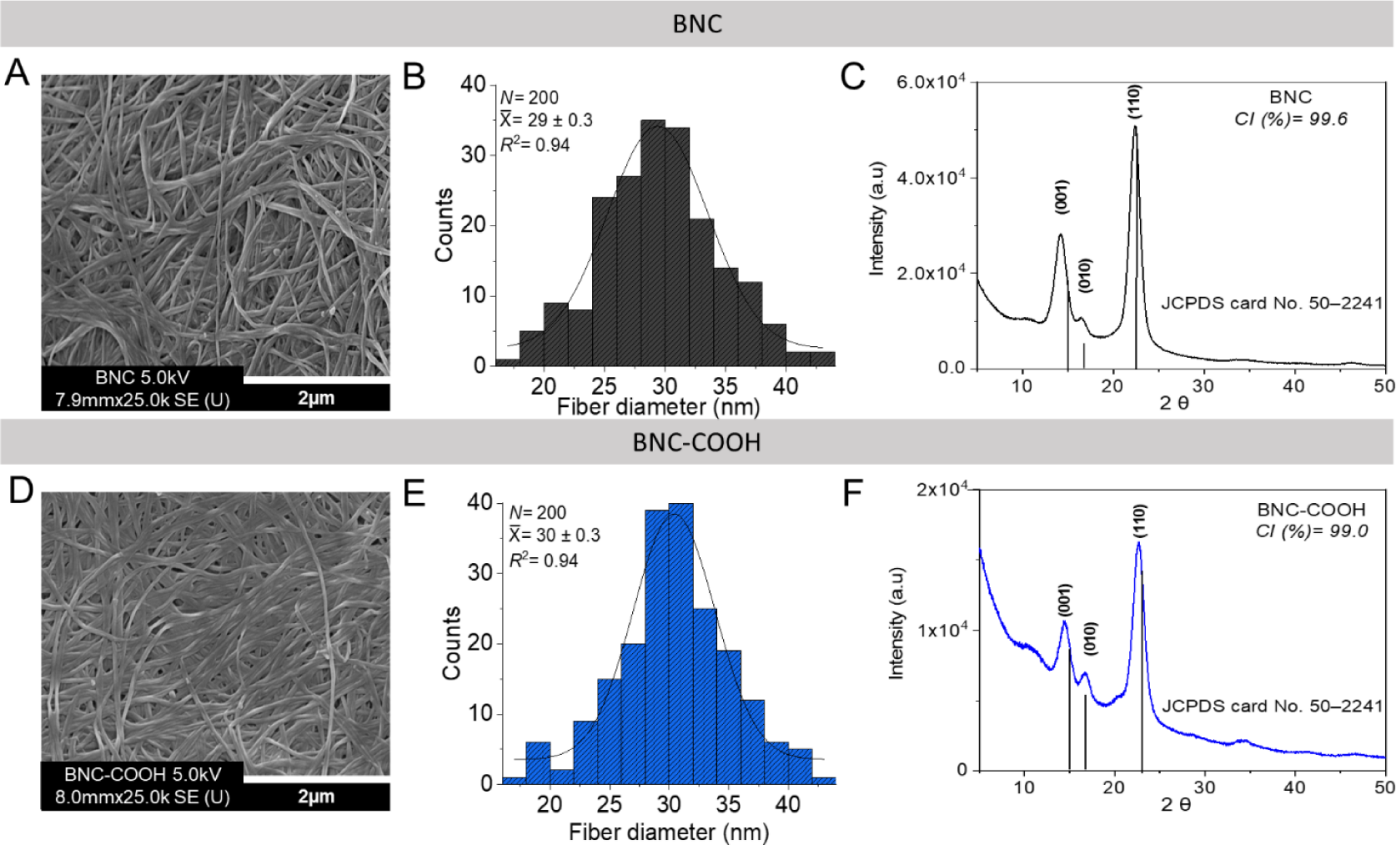
Morphology, size, and crystallinity of native BNC and BNC–COOH. (A and D) HR-SEM micrography of BNC and BNC–COOH, respectively (scale bar = 2 μm). (B and E) The fiber diameter of BNC and BNC–COOH was calculated using ImageJ software. (C and F) XRD pattern and crystallinity index (CI %) calculated of BNC and BNC–COOH.

The morphological analysis of the nanofibrils is presented in Figure 2, which shows the length and diameter histograms of BNC and BNC–COOH obtained by SEM analysis processed with ImageJ. The morphology (Figure 2A and D) and nanofibril size (Figure 2B and E) of the native BNC and carboxylated BNC remain unchanged following the functionalization with carboxylic acids using oxalic acid and choline chloride-based DES. There is no evidence of fibrils’ rupture, thus confirming that the functionalization occurred on the BNC surface.^7^

BNC is a biopolymer characterized by higher crystallinity compared to wood-based and plant-based cellulose.^31^ Unlike plant-derived cellulose, BNC is not associated with other polymers, such as lignin and hemicellulose, due to its unique biosynthetic process.^32^ BNC is classified as a subpolymorph of Iα cellulose,^31^ featuring a triclinic unit cell, which is characteristic of most algal and bacterial cellulose.^33^ The crystallinity in the structure of BNC confers attractive physicochemical properties, such as density, Young’s modulus, and tensile strength, which are analogous to those of collagenous fibers in bone tissue and extracellular matrix. Crystallinity is a key aspect involved in promoting cell adhesion, cell proliferation, and differentiation in nanocellulosic biomaterials.^17,32^ The X-ray diffraction (XRD) analysis of both native and functionalized nanocellulose assessed the changes in the crystalline structure of BNC resulting from the DES treatment. The XRD pattern showed a crystallinity index (CI %) of 99.6 for native BNC and 99.0 for the functionalized BNC–COOH, calculated by the Segal method^34^ (Figure 2C and F). These results suggest that functionalization occurred on the BNC surface, likely on the crystalline plane (001) due to the introduction of carboxylic acids at the C-6 position of the cellulose ring, as discussed above. Surface functionalization is critical for maintaining the BNC fibrils’ features while enhancing the bionanointerface with cells. Surface functionalization of BNC using the complete ChCl-OA-based reactive DES enhanced its surface chemistry while preserving its key mechanical and morphological properties.^35^

The hydrophilicity of BNC can be enhanced by introducing polar and ionizable groups, such as carboxylic acids,^32^ through the incorporation of multiple water molecules, improving its dispersibility in water, facilitating water uptake and hydrogel formation. Therefore, the surface carboxylation of BNC significantly influences the water absorption capacity of the hydrogel-forming fibrils, making it highly suitable for 3D cell culture applications. Not only has surface chemistry been reported to affect cell adhesion and differentiation,^17^ but also wettability, surface rugosity, and surface energy play important roles.

**Figure 3:**
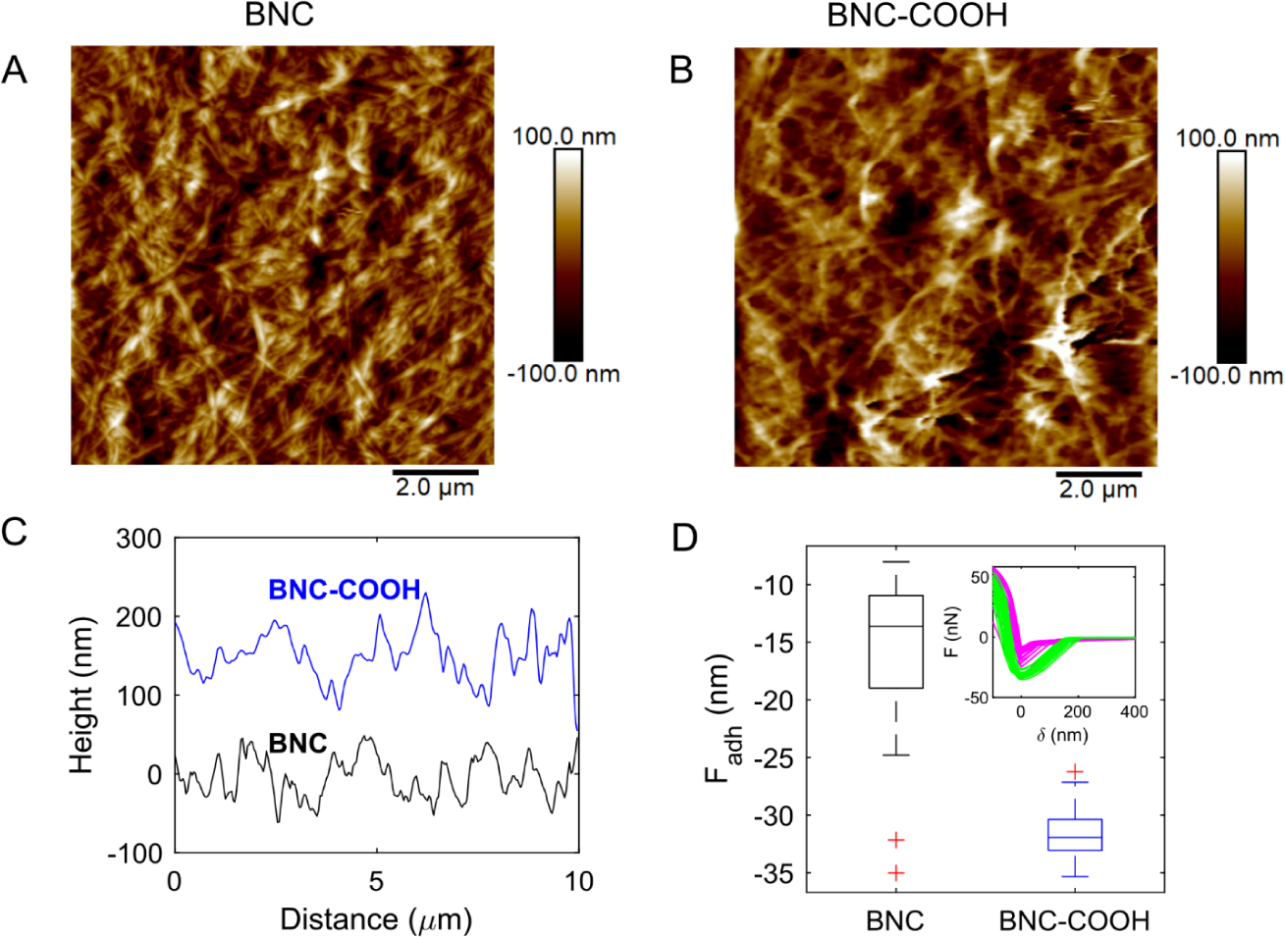
AFM surface morphology and force adhesion of native BNC and BNC–COOH. (A and B) Morphology of native BNC and BNC–COOH, respectively. (C) Horizontal profiles were obtained in the middle of the morphology images. (D) Quantitative distribution of adhesion force data measured between the AFM-tip and the surface of dried BNC and BNC–COOH; inset shows the *F*–δ curves.

To gain deeper insights into the surface energy modification of BNC following functionalization with carboxylic acids, surface morphology, and stress–strain (*F*–δ) curves were obtained by using AFM (Figure 3). The surface morphology of BNC displays more fibril agglomeration and compaction than BNC–COOH, with a root-mean-square (RMS) roughness of 30 ± 0.13 nm for BNC and 34 ± 0.24 nm for BNC–COOH (Figure 3A and B). The surface morphology for native BNC without chemical modification is similar to that observed by Jabbour et al.^7^ AFM height profiles revealed that BNC–COOH shows a high surface roughness (Figure 3C), which is induced by surface chemical functionalization. The effect of COOH on the BNC was further evaluated through the adhesion force (*F*_adh_) determined from the *F*–δ curves (Figure 3D). The adhesion value by AFM has been used to determine the number of significant adhesion force events by the cells or the substrate and the forces required to break each adhesion bond.^36^ The *F*_adh_ ratio for BNC–COOH to BNC was 2.34, indicating a higher force to detach the probe from the BNC–COOH surface. To understand the increase in *F*_adh_, it is necessary to consider that the SiO_*x*_ covering the AFM probe tip contains ∼5 −OH nm^–2^ terminations at ambient conditions.^37^ These groups can form water monolayers, even at low relative humidity levels. Thus, the *F*_adh_ encloses information from the tip–sample interaction, which is governed by Van der Waals and capillary forces resulting from water condensation. This result is a hydrophilic interaction between the AFM tip and BNC–COOH due to the presence of hydroxyl groups. Thus, the enhanced physical and chemical properties measured by the *F*_adh_, along with the surface morphology, contribute to the BNC–COOH functioning as a scaffold for cell proliferation, as will be demonstrated in the following sections.^17,38^

### Water Uptake of BNC and BNC–COOH

**Figure 4:**
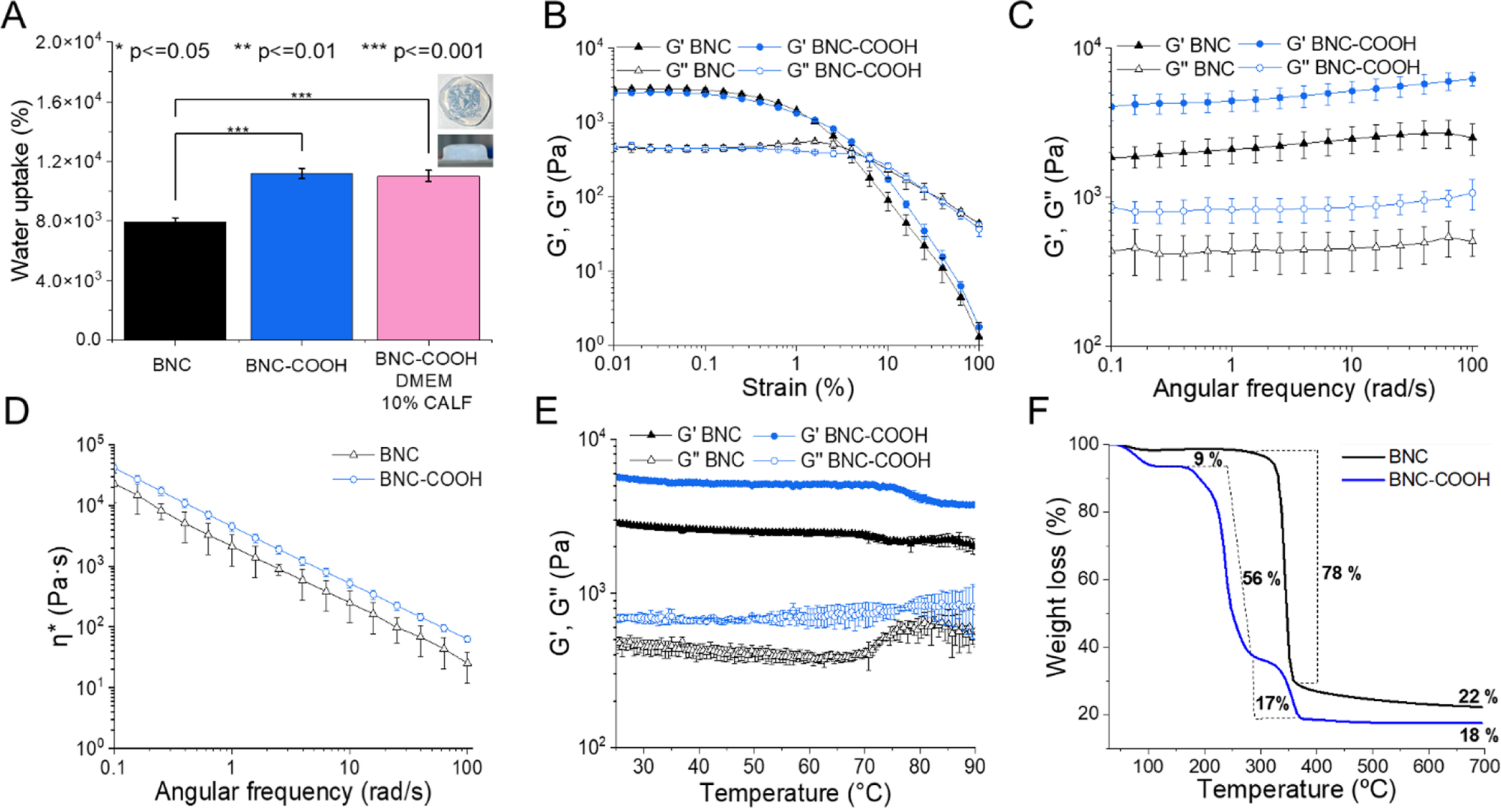
(A) Water uptake of BNC, BNC–COOH, and BNC–COOH with DMEM 10% CALF hydrogels and viscoelastic properties at 25 °C and the images of BNC–COOH hydrogels with deionized water. (B) Strain sweep, (C) frequency sweep tests (*G*′, represented by solid symbols; *G*″, by open symbols, plotted against frequency); (D) complex viscosity test (η*), (E) temperature sweep. (F) Thermal properties of BNC of BNC–COOH hydrogels. All tests were conducted in triplicate.

Water uptake is a crucial characteristic of cellulosic materials, as it reflects their surface properties and determines their ability to form hydrogels. Herein, after thoroughly washing out the DES used to functionalize the BNC, the dry BNC and BNC–COOH were used for further studies. Figure 4A illustrates the water uptake capacities of BNC and BNC–COOH when in contact with water to form hydrogels at equilibrium. It was found that the BNC hydrogels exhibited significant swelling within just 1 h of immersion in an excess of water, after which there was a negligible increase in water absorption with additional immersion time. At equilibrium, the BNC hydrogels absorbed a maximum of 7,917 ± 242% water, while the BNC–COOH hydrogels demonstrated a significantly higher water uptake of 11,180 ± 353% after functionalization (*p* < 0.001). Additionally, the hydrogels exhibited good optical transparency and excellent water absorption capabilities (Figure 4A). These conditions are optimal for cell culture and confocal microscopy analysis.^39^ Regarding these findings, Smyth et al.^40^ reported the impact of hydration of cellulose nanofibril (CNF) thin films on stem cell culture. They reported a maximum water uptake percentage of 13% for the CNF materials. Similarly, Yang et al.^41^ reported a swelling ratio of 5.5% for a BNC hydrogel with dimensions of 2 × 2 cm, demonstrating the importance of surface functionalization of cellulose nanofiber with hydrophilic groups.

In this research, the water in the BNC–COOH hydrogel was replaced with Dulbecco’s Modification of Eagle’s Medium (DMEM) culture media containing 10% calf serum, while maintaining the maximum swelling (11,019 ± 164%) observed in the BNC–COOH. Similarly, Rasheed et al.^42^ reported that nanocellulose fiber scaffolds retained their structure in DMEM cell culture media, with maximum swelling achieved after 24 h of immersion. Additionally, cell culture media contain ions, amino acids, and various proteins necessary for cell growth, which can enhance swelling and support the hydrogel structure. Moreover, the ions in cell culture media may contribute to the hydrogel formation through ionic cross-linking. For instance, Curvello and Garnier^43^ designed a nanocellulose hydrogel and demonstrated that cationic cross-linking supports cell adhesion and intestinal organoid formation. Gao et al.^44^ found that DMEM cell culture significantly increased the swelling of a hydrogel based on the self-cross-linking of phenylboronic and hyaluronic acid. Basic amino acids also stabilize the hydrogel, as they carry a positive charge under physiological conditions (pH 7.4). Furthermore, Li et al.^45^ stated that DMEM contains a variety of ions that can promote potential intermolecular interactions, enhancing mechanical resistance.

Considering the above results, to further study the feasibility and properties of BNC–COOH hydrogels for cell culture, hydrogels composed of 0.1 wt % of both BNC and BNC–COOH were employed, which are within the range of stable hydrogels found in the swelling test (Figure 4A), *i.e.*, hydrogels that retained a stable physical form without leaking solvent at room temperature for 4 h.

### Rheological Properties of the Hydrogels

Hydrogels were characterized mechanically in the linear viscoelastic region (LVR). The LVR is defined as the deformation range where the elastic and loss moduli (*G*′ and *G*″, respectively) are independent of deformation (γ%). After a critical deformation (γ%), *G*′ and *G*″ exhibit a sharp change in their slope, diminishing as a function of the deformation, which is indicative of the breakdown of the hydrogel microstructure. To measure the LVR for hydrogel samples, strain sweeps were performed from 0.01 to 100% at a constant frequency (*f*) of 1 Hz and a temperature of 25 °C. Figure 4B shows *G*′ and *G*″ as a function of deformation for BNC and BNC–COOH hydrogels. Results indicate that both hydrogels depict a similar trend with LVR values γ% ≤ 0.2.

Figure 4C shows *G*′ and *G*″ as a function of the frequency sweep experiments for BNC and BNC–COOH samples. The applied strain for two hydrogels was 0.05%, which is within the linear viscoelastic region and at a temperature of 25 °C. The elastic and viscous moduli for both hydrogels slightly augment with increasing frequency, but they are overall relatively constant. Such rheological behavior indicates the presence of permanent junction points as opposed to transient entanglements, leading to a viscoelastic plateau.^46^ The value of *G*′ for the BNC–COOH sample is higher than that for BNC. This may be attributed to the formation of denser internanofiber interactions at higher frequencies related to the surface carboxyl moieties in BNC–COOH.^47^ Besides, the elastic modulus is consistently larger than the loss modulus (*G*″), indicating that the hydrogels behave as gel-like over the frequency range studied. On the other hand, the relative relationship tan δ = *G*″/*G*′ (dissipation capacity) is similar for both samples, with tan δ = 0.17.

Figure 4D illustrates the relationship between the modulus of the complex viscosity (η*) and the angular frequency for the samples. The complex viscosity for BNC and BNC–COOH diminishes as frequency increases, as described above, and the BNC–COOH sample shows the highest viscosity, while the BNC sample is two times less viscous. The BNC–COOH increased viscosity is attributed to the formation of hydrogen bonds resulting from the surface functionalization with carboxylic acids.^48^

Finally, Figure 4E shows the temperature dependence of *G*′ and *G*″ for BNC hydrogels at a frequency of 1 Hz. The temperature was increased from 25 to 100 °C at a heating rate of 5 °C min^–1^. The storage modulus (*G*′), representing the solid component of the rheological behavior, remains constant up to 70 °C for BNC and 77 °C for BNC–COOH, after which it declined for both materials. This effect is more pronounced in BNC hydrogels than in BNC–COOH hydrogels, demonstrating greater resistance to temperature and water loss. These results indicate that the structure of the BNC–COOH is physically more stable at higher temperatures compared to previously reported nanocellulose hydrogels, which exhibited limited stability above 65 °C.^47^ This increased stability is attributed to the enhanced interactions among the individual functionalized nanofibers.

### Thermal Properties of BNC and BNC–COOH

The thermal properties of BNC and BNC–COOH were assessed through thermogravimetric analysis (TGA) to corroborate the increase in the number of carboxylic acid groups after esterification. The sample’s thermal degradation behaviors can be divided into two processes for BNC (Figure 4F). The initial mass loss occurs in the 30–150 °C range, corresponding to the evaporation of residual and unbound water in the BNC. Significant differences in the thermal degradation of the two samples (BNC and BNC–COOH) were observed during this weight-loss stage. For BNC, the pyrolysis started between 300 and 360 °C, with the thermal decomposition of shorter chains and amorphous cellulose.^49^ During this process, the glycosidic linkages in cellulose were cleaved, producing H_2_O, CO_2_, alkanes, and other hydrocarbon derivatives, which contributed to the rapid drop in the TGA curves^50^ and resulted in a weight loss of 78%. These findings are consistent with previous reports.^35,51^ In contrast, the pyrolysis of the BNC–COOH sample continued in the range of 200–300 °C, resulting in a 56% weight loss, primarily associated with the pyrolysis of cellulosic materials. The weight loss of 17% in BNC–COOH in the range of 300–400 °C corresponds to the decomposition of hydroxyl and carboxylic acid groups.^52^ After 400 °C, the cellulose thermal degradation was nearly completed, leading to the carbonization stage. The final residue of BNC at 700 °C was 22%, while that of BNC–COOH remained relatively lower, 18% of the initial mass.

Collectively, the composition and morphological and physicochemical properties of the functionalized BNC demonstrate the formation of stable hydrogels that can be explored to sustain their use as scaffolds for cell culture (Movies S2 and S3). Besides examining the chemical and physical structural aspects of BNC, this study focused on how mammalian cells interact with the material. Mouse fibroblast cells (3T3-L1) were chosen for their ability to grow in a 3D cell culture, making them particularly relevant for research on materials intended for tissue engineering applications.^53,54^ Preadipocytes (3T3-L1) were cultivated on BNC and BNC–COOH to assess the biomaterial’s capacity to support cell attachment, anchoring, growth, and differentiation.

### Biocompatibility of Hydrogels

**Figure 5:**
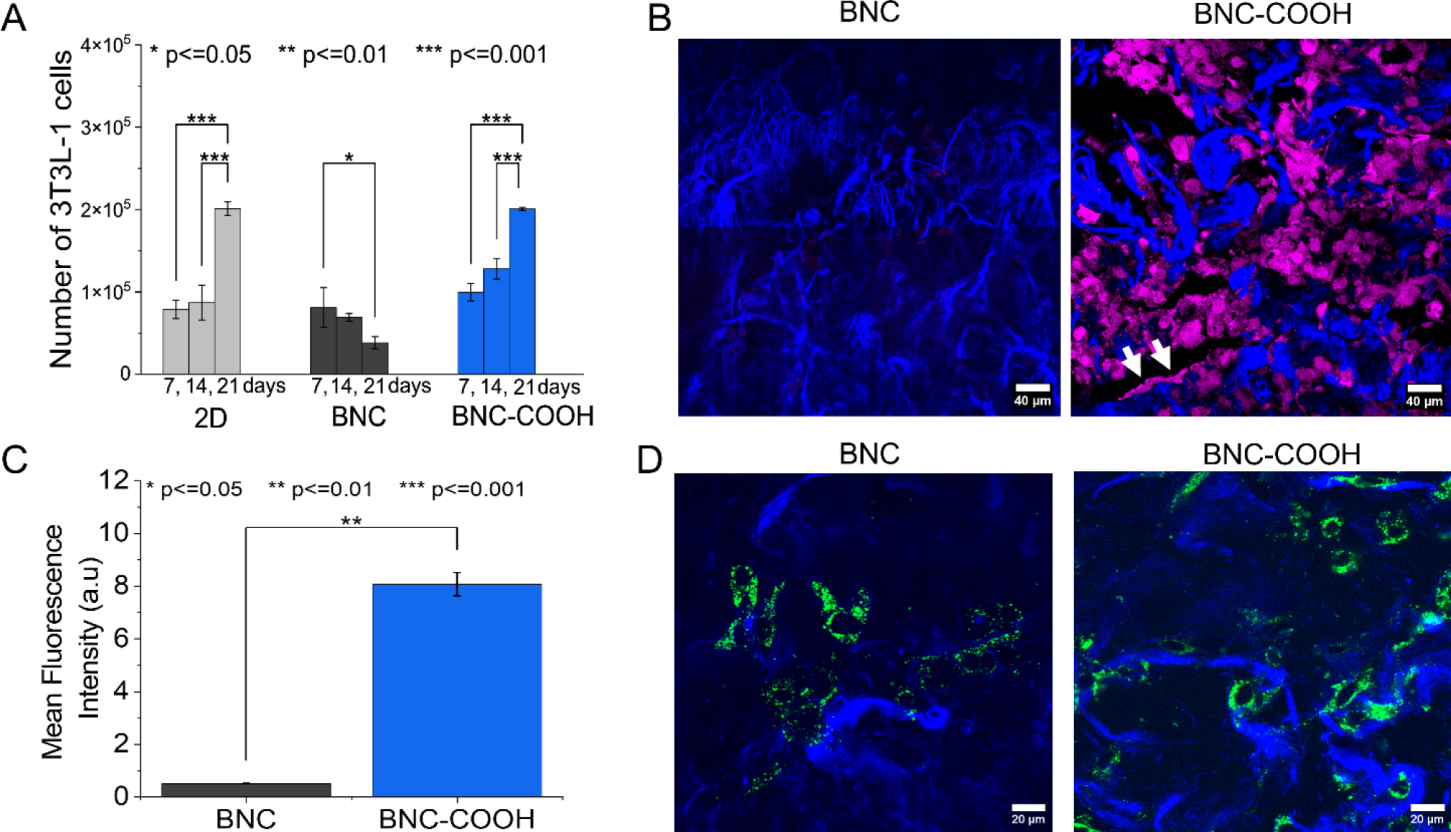
Biocompatibility of BNC and BNC–COOH hydrogels. (A) Cell viability of 3T3-L1 fibroblasts in BNC and BNC–COOH hydrogels over 7, 14, and 21 days. (B) Confocal microscopy micrographs of the 3T3-L1 cells cultured in BNC and BNC–COOH hydrogels at 21 days (scale bars represent 40 μm). Cells were stained with resorufin (pink color), while bacterial nanofibers were stained with calcofluor (blue color). (C) Mean fluorescence intensity of the DCFDA (shown in green in D) for quantifying ROS production in cells grown in hydrogels. (D) ROS production in 3T3-L1 cells at 21 days. Cells are green in color due to the fluorescence of DCFDA, and the bacterial nanofibers are blue due to the staining with calcofluor (scale bars represent 20 μm). The bars represent the mean ± standard deviation. Statistical significance is as follows: *p < 0.05; **p < 0.01; ***p < 0.001.

Hydrogels are the most widely used 3D matrices for cell culture due to their high biocompatibility and fluid-retaining structure.^55^ In this study, we assessed the biocompatibility of BNC and BNC–COOH hydrogels for culturing 3T3-L1 fibroblast cells in a 3D arrangement. As shown in Figure 5A, the BNC hydrogel exhibited low biocompatibility, resulting in limited growth of 3T3-L1 cells over 7, 14, and 21 days. The growth of fibroblast 3T3-L1 in a 2D arrangement on a polystyrene Petri dish reached a maximum cell count of 201,079 ± 8,423 cells at 21 days. Beyond this time, saturation of the Petri dish impeded further cell growth, underscoring that 2D platforms are unsuitable as long-term platforms. However, after 21 days, approximately 38,390 ± 7,316 3T3-L1 fibroblasts were found on the BNC scaffold, whereas around 200,778 ± 1,863 cells were present on the BNC–COOH hydrogel scaffold. This result indicates that the cell growth on the BNC–COOH scaffold was significantly enhanced, being 7.3 times higher than that on the BNC hydrogel. Given the improved cell growth on the BNC–COOH hydrogel scaffold, we extended the observation period to 40 days. In this regard, it has been reported that BNC scaffolds effectively support cell ingrowth and facilitate subsequent cartilage remodeling in joints, where BNC hydrogel underwent 3D laser perforation before cell culture.^56^

However, upon measuring the number of cells on the scaffolds at 40 days, we found that the counts were significantly lower in both cases compared to measurements taken on day 21. Resazurin perfusion within the scaffold was likely hindered due to the high cell density, as observed in the confocal microscopy micrographs, a phenomenon previously reported.^57,58^ Consequently, we analyzed cell growth using confocal microscopy, as shown in Figure 5B. The confocal microscopy images in Figure 5B reveal a significantly higher cell density in the BNC–COOH hydrogel compared with the BNC hydrogel. Additionally, it is possible to observe the long and thin filopodia of 3T3-L1 cells, which facilitate anchoring to hydrogel nanofibrils, as indicated by the white arrows in Figure 5B (BNC–COOH). The −COOH functional groups on the BNC scaffold enhance the adhesion capacity of 3T3-L1 cells observed in the BNC–COOH hydrogel. These results are consistent with findings from mesenchymal stem cells cultured on BNC hydrogels, where optimal attachment is evident through their distinctive filopodia extensions.^16^

Reactive oxygen species (ROS) are byproducts of cellular respiration and serve as quantifiable parameters related to cell proliferation. In Figure 5C, ROS production at day 21 days of culture is represented as the mean fluorescence of DCFDA obtained from the confocal micrographs of 3T3-L1 cells (Figure 5D). Fibroblast cells cultured in BNC–COOH exhibit higher ROS production compared to those in BNC, which display moderate ROS levels. This increased ROS production is consistent with the increase in the 3T3-L1 cell number observed after 21 days of growth in the BNC–COOH hydrogel. Therefore, moderate ROS production correlates with the proliferation of 3T3-L1 cells cultured in the BNC–COOH hydrogel. This proliferation may represent an adaptive response to hypoxia, during which cells activate their antioxidant systems, including antioxidant enzymes such as superoxide dismutase, catalase, and glutathione-dependent mechanisms.^58^ These systems help mitigate the toxic effects of elevated ROS levels, enhancing proliferative capacity.^59^ Furthermore, it has been reported that the cell cycle is coupled to ROS production oscillations.^60^

As shown above, BNC–COOH enhanced cellular proliferation in a time-dependent manner, proving to be a suitable 3D scaffold. Thus, as an additional property of the arrangement of the nanocellulose fibers, it was interesting to assess whether the 3D architecture of the cells would induce their maturation. In this sense, 3T3-L1 cells are an excellent model to test preadipocyte maturation, with a fibroblast-like morphology to mature adipocytes characterized by lipid deposition in intracellular droplets.

**Figure 6:**
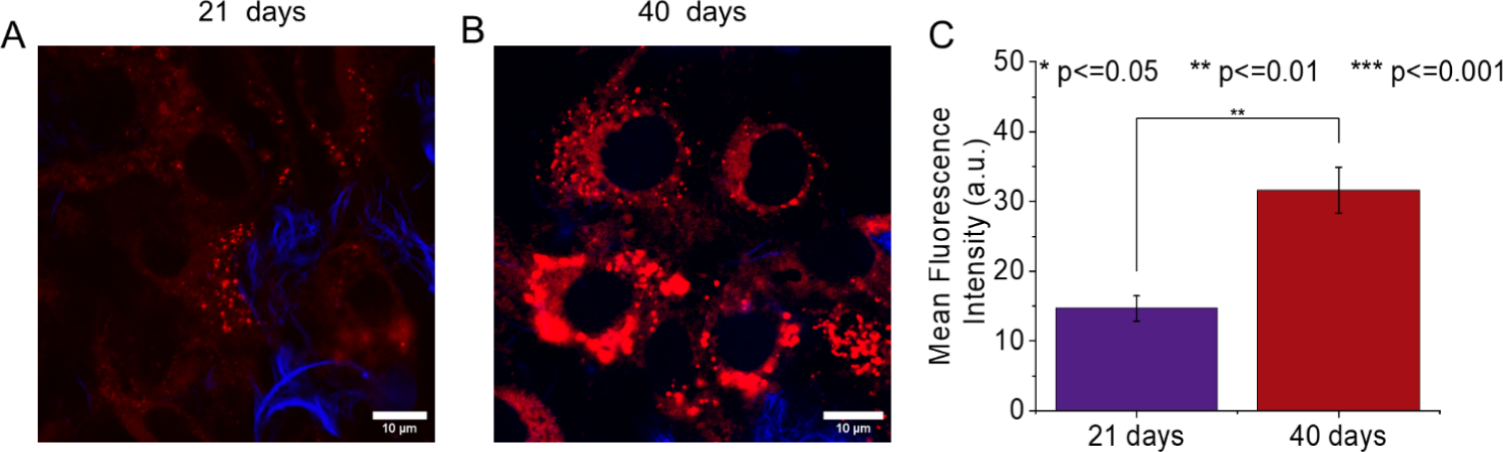
Differentiation of 3T3-L1 cells cultured in the BNC–COOH hydrogel. Confocal microscopy images of 3T3-L1 cells (red color) stained with Nile red, along with bacterial nanofibers (blue color) stained with white of calcofluor at (A) 21 and (B) 40 days of culture. (C) Lipid deposition in intracellular droplets measured by mean fluorescence intensity from confocal micrographs. Scale bars represent 10 μm. The intense red spots correspond to lipid droplets, which indicate cell maturation.

The results presented in Figure 6A,B indicate that the 3D structure formed by BNC–COOH and the supportive hydrogel environment contribute to the differentiation of preadipocytes into mature adipose cells at 21 days. Additionally, there is a significant increase of 2.2-fold in lipid droplet production in the 3T3-L1 cells at 40 days (Figure 6C), accompanied by shortened filopodia and more rounded morphology. Similar findings have been reported in Matrigel with DMEM, indicating that the maturation of 3T3-L1 cells can take between 2 and 5 weeks.^61^ During the differentiation process, 3T3-L1 cells not only change into a more spherical morphology but also begin to accumulate lipids and express specific markers of fat differentiation^62^ (Figure S3). A key morphological characteristic indicative of differentiation is the presence of lipid droplets, which can be stained with various fluorophores, such as Nile red. Furthermore, it has been shown that 3D cell culture promotes adipogenesis similar to that observed in an *ex vivo* model.^63^ In this work, adipogenesis is primarily attributed to the carboxyl functional groups on bacterial fibrils and the stiffness of the hydrogel formed. Similarly, Rasha et al.^17^ reported that an increase in hydroxyl groups on the surface of CNF samples promotes osteogenic differentiation and enhances the biological performance of scaffolds for bone tissue regeneration. These findings correlate with the surface characteristics of BNC–COOH, which has a high content of hydroxyl and carboxyl functional groups in its chemical structure, supporting cell proliferation.^38^ Additionally, Malandain et al.^1^ designed a hydrogel composed of collagen type I and bacterial nanocellulose fibers, resulting in a 43% increase in hydrogel stiffness due to the combination of these natural polymers.

Furthermore, studies have reported that culturing 3T3-L1 cells in 3D environments, such as hydrogels and spheroids, promotes adipogenesis, accompanied by an increased expression of mitochondrial genes, and key metabolic phenotypes associated with adipocyte maturation. This contrasts with preadipocytes grown in a 2D monolayer.^64^ Consistent with these findings, our results demonstrate that the 3T3-L1 cells proliferated and matured within the 3D structure provided by the BNC–COOH hydrogel.^65^ Its physicochemical and rheological properties support enhanced cell proliferation, while the nanofibers within the hydrogel facilitate cell anchorage. Additionally, the chemical composition of bacterial nanocellulose and its 3D arrangement promote cell maturation, making carboxylated BNC scaffolds an effective platform for 3D cell culture.

## Conclusion

In this study, we developed a sustainable method to introduce carboxylic moieties onto bacterial nanocellulose fibers, forming hydrogels through a nonaqueous deep eutectic solvent. The resulting BNC–COOH hydrogel has a high concentration of carboxylic acid functional groups, enhancing swelling, viscosity, surface roughness, and adhesion. It also demonstrates greater resistance to deformation, reduced water loss, and improved thermal stability compared to native BNC. This hydrogel demonstrated biocompatibility with preadipocyte 3T3-L1 cells and exhibited exceptional capability in promoting cell growth. Our findings indicate that the biomimetic micronano features on BNC–COOH are significantly more effective than those found in native BNC environments, providing an optimal bionanointerface for cell anchorage. As a result, the BNC–COOH hydrogel promotes outstanding cell growth. It mediates the maturation of preadipocytes into mature adipocytes, as evidenced by a decrease in extended filopodia and a rounded cell morphology, along with increased content of intracellular lipid droplets after 40 days of culture, as demonstrated by confocal microscopy images. The maturation of 3T3-L1 cells was facilitated by carboxylic acid functional groups, and the 3D structure was provided by the bacterial nanofibers in the hydrogel. This microenvironment promotes cell growth, allowing cells to interact in 3D arrangements, which enhances cellular communication. Thus, we demonstrated that a reactive DES based on oxalic acid and choline chloride serves as an effective method for creating biocompatible 3D hydrogel scaffolds with enhanced physicochemical characteristics and improved biological functionality for 3D cell culture applications.

## Experimental Section

### Materials

Bacterial nanocellulose was purchased from Nano Novin Polymer Co. (Sari, Iran), and oxalic acid dihydrate (ACS reagent ≥99% purity) and choline chloride (ACS reagent ≥99% purity) were purchased from Sigma-Aldrich. Deionized water (18 MΩ) was used in all of the experiments. Phosphate-buffered saline (PBS), Dulbecco’s Modified Eagle’s Medium-high glucose (DMEM), antibiotic-antimycotic solution (10000 U/mL penicillin and 10 mg/mL streptomycin), l- glutamine, sodium bicarbonate, and calf bovine serum (CS) were purchased from Sigma-Aldrich Chemical Co. (St. Louis, Missouri, USA).

### The Hydrogel of Bacterial Nanocellulose

The commercial BNC is supplied as a large sheet measuring 210 mm in width, 297 mm in height, and 1 mm in thickness. The sheets were cut into discs with a diameter of 2.5 cm for the rheology tests and 1 cm for the rest of the studies. Also, BNC is stored in 1% sodium hydroxide (NaOH). It was rinsed with deionized water at 60 °C for 4 h, with water changes every 30 min, until a pH of 7 was reached to confirm the removal of NaOH. Washing the BNC with deionized water promoted swelling, leading to the formation of the final hydrogel.

### DES Preparation

First, the choline chloride was oven-dried before being used to remove trace water. Then, the dry choline chloride was mixed with the oxalic acid dihydrate at 60 °C until the eutectic mixture was obtained in a 1:1 molar ratio.

### Bacterial Nanocellulose Hydrogel Esterification

BNC hydrogels of 1 cm diameter were immersed in the eutectic solution of oxalic acid dihydrate and choline chloride for 1 h at 25 °C to allow the exchange of deionized water by the DES. Subsequently, the hydrogels with DES were lyophilized for 24 h. The eutectogels were immersed in the eutectic mixture at 60 °C for 1, 2, and 3 h. After that time, the eutectogels were washed with 20 mL of deionized water, and the conductivity of the solvent was measured with an EC meter (HI-2030, HANNA Instruments, USA) between each wash to ensure the removal of DES (≤18 μS cm^–1^). Finally, the BNC–COOH hydrogels functionalized with carboxylic acid groups were preserved in deionized water before future use. Notably, the BNC hydrogels were not dried out for functionalization with carboxylic acid groups; only the water was exchanged for DES to maintain the swelling levels.

### SEM, ATR-FTIR, and XRD of BNC and BNC–COOH

The hydrogels were lyophilized for SEM, ATR*-*FTIR, and XRD characterization. SEM images of the BNC and BNC–COOH were obtained by using a high-resolution scanning electron microscope (HR-SEM) model Hitachi SU8230. The samples of BNC and BNC–COOH were gold-coated by vacuum deposition. The vibrational states of COOH in BNC were investigated using Fourier transform infrared spectroscopy on a PerkinElmer Spectrum Two equipped with an ATR (attenuated total reflectance) accessory with a diamond crystal. The spectra were taken with a 2 cm^–1^ spectral resolution in the 600–4000 cm^–1^ range. The XRD of BNC and BNC–COOH was scanned at 0.5 °C min^–1^ on a Rigaku Ultima IV diffractometer, operating at 35 kV, 15 mA with CuKα radiation λ = 1.5406 Å on a 2θ scale with a step size of 0.02° with a DTex detector. The crystallinity index (CI %) of dried nanocellulose was determined using the following Segal equation:^34^
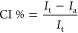
1where *I*_t_ is the intensity of the (110) peak at 22.7° 2θ, and *I*_a_ is the intensity of amorphous cellulose at 18° 2θ (eq 1).

### Surface Morphology and Adhesion Force of BNC and BNC–COOH

Morphology and adhesion force measurements of BNC and BNC–COOH lyophilized samples were performed using a Bruker Dimension Edge atomic force microscope (AFM) operating at a temperature of ∼25 °C and a relative humidity of ∼55%. The measuring probe was a model SNL-10 with nominal values of a 0.24 N/m spring constant and 56 kHz free resonance frequency. Twenty-five force–separation (*F*–δ) curves were acquired at arbitrary locations within a 10 μm × 10 μm area, from which the adhesion force (*F*_adh_) was obtained. The sensitivity calibration was performed on a sapphire sample, while the spring constant was obtained by using the thermal noise method included in the AFM apparatus. The samples were mounted on a glass slide and taped at the ends. No special treatment, such as intentional wetting or drying was performed on the specimen surface during the measuring to avoid bias in morphology measurements or *F*–δ curves. Thus, the specimens can be considered dried in the laboratory environment. Each AFM morphology image contains 512 pixels ×512 pixels, and a horizontal profile in the middle of the image was obtained for comparison, i.e., on line 256. Root mean square roughness (Rq) was calculated for each AFM morphology image.

### Water uptake study

The hydrogel samples used for the water absorption study were circular discs with a diameter of 10 mm and a dry weight of 3.56 ± 1.15 mg, immersed in an excess of deionized water at room temperature. Three samples of each composition were used. The samples were removed from the water and placed on blotting paper, with a second sheet placed on top of the sample. The samples were weighed to determine the amount of water uptake at 30 min intervals over 4 h. The water uptake (WU) was determined according to the following equation:
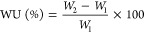
2where *W*_1_ is the weight of the BNC hydrogel before immersion, and *W*_2_ is the weight of the BNC hydrogel after immersion (eq 2).

### Rheology

The rheological properties of the BNC and BNC–COOH were assessed with an Anton Paar Rheometer MCR-702 with a parallel plate geometry of 25 mm diameter, and the samples used were hydrogels of circular form with a diameter of 25 mm. The discs were cut from a large sheet (210 mm width, 297 mm height, and 1 mm thickness) of the as-received BNC. Then the samples were washed with deionized water for 4 h at 60 °C to remove NaOH. In this process, the BNC fibers are swollen with deionized water to form the hydrogel. Samples were loaded onto the bottom plate of the rheometer, with a gap of 3.3 mm between the sample and the bottom base plate. All measurements were conducted at a constant temperature of 25 °C in a covered area of the rheometer to prevent the sample from drying out and to ensure a consistent temperature throughout the measurements. The samples were subjected to various measurement profiles. To determine the linear viscoelastic region (LVR), strain sweep experiments were performed from 0.01 to 100% at a frequency of 1 Hz and 25 °C. A dynamic frequency sweep test was performed from 0.01 to 100 rad s^–1^ to determine the dynamic storage modulus (*G*′) and loss modulus (*G*″) of each hydrogel at a strain rate confirmed to be within the linear viscoelastic range (LVR) for each hydrogel. All measurements were performed at 1 Hz with 0.05% strain. To study the thermal evolution of the systems, temperature sweep experiments were carried out with frequency set within LVR, and temperature was increased from 25 to 100 °C at a constant rate (5 °C min^–1^). Three repeated measurements were performed for each formulation, and mean values were reported.

### Thermal Properties of BNC and BnC–COOH

To determine the thermal stability and decomposition pattern of nanocellulose, thermogravimetric analysis (TGA) was performed in a Mettler Toledo TGA-DSC 2. The temperature range for the analysis of two samples (BNC and BNC–COOH) was set between 25 and 700 °C with a heating rate of 10 °C min^–1^, and a nitrogen atmosphere with a 40 mL min**^–1^** flow rate was used.

### Cell Culture

The fibroblast cell line (NIH-3T3-L1) was obtained from the American Type Culture Collection (ATCC; Manassas, Virginia, USA). The cells were cultured and maintained in DMEM supplemented with 10% calf serum (CS), 1% penicillin/streptomycin (v/v), 1% l-glutamine (v/v), and 2.5 g/L sodium bicarbonate. The cell line was propagated and maintained following ATCC’s recommendations. Cultures were kept in an incubator at 37 °C under a humidified atmosphere of 5% CO_2_. The hydrogels were sterilized with UV irradiation for 15 min and immersed in DMEM with 10% calf serum overnight at 4 °C. Before cell cultivation, the hydrogels were tempered at 37 °C. After tempering the BNC and BNC–COOH hydrogels, each hydrogel was placed in a well of a 24-well plate. Then, 1.5 mL of calf-serum-supplemented DMEM containing 30,000 cells was carefully added to cover the surface of each hydrogel completely. The hydrogels were then incubated at 37 °C under a humidified atmosphere of 5% CO_2_.

### Cell Proliferation Assay

Cell proliferation was assessed by reducing resazurin (blue) to resorufin (pink) due to the high metabolic activity of living cells, which serves as a monitor for cell viability. The reduction of resazurin can be detected through absorbance readings of the ratio of resorufin/resazurin at 570/600. 30,000 cells were cultivated in the hydrogels of BNC and BNC–COOH for 7, 14, and 21 days. For the resazurin assay, the cells were incubated for 24 h with 10% resazurin, and the absorbance was measured by a UV–vis spectrophotometer (Genesys 840-208200, Thermo Scientific, USA). The number and fluorescence of cells grown on the BNC scaffolds were calculated as previously reported by our group, following a standard curve that correlates the number of cells with their corresponding percentage reduction of resazurin.^65^ Micrographs of 3T3-L1 cells grown on the hydrogels were obtained by a Zeiss LSM880 confocal laser scanning microscope using an argon laser with a 63× immersion oil objective.

Micrographs of 3T3-L1 cells were obtained by a Zeiss LSM880 confocal laser scanning microscope using an argon laser with a 63× immersion oil objective.

### ROS Assay

Cells (30,000 per hydrogel) were cultured in BNC and BNC–COOH hydrogels for 21 days. The ROS levels were measured in preadipocytes 3T3-L1 using 2,7-dichlorofluorescein diacetate (DCFH-DA; Cat. No.: D6883, Sigma-Aldrich, St. Louis, Missouri, USA), which is converted into the fluorescent 2,7-dichlorofluorescein (DCF) in the presence of ROS. The cells were incubated with 25 μM DCFH-DA for 30 min. Then, cells were rinsed with PBS, and ROS level production was detected by a Zeiss LSM880 confocal laser scanning microscope using a HeNe543 laser with a 63× immersion oil objective. The entire process was performed in the dark.

### Nile Red Staining

Thirty thousand cells were cultured in BNC and BNC–COOH hydrogels for 21 days and only in BNC–COOH hydrogels for 40 days. The 3T3-L1 cells did not undergo chemical differentiation induction before the experiments. Cells were incubated with 18 μM Nile Red for 15 min, with the entire process carried out in the dark. The fluorescence was detected by a HeNe633 laser with a 63× immersion oil objective in a Zeiss LSM880.

### Staining of Bacterial Nanocellulose Fibers

The bacterial nanocellulose fibers were stained with 0.1% calcofluor white for 15 min at 37 °C in a humidified atmosphere with 5% CO_2_. The hydrogel was then carefully washed three times with PBS (pH 7.4). Following staining, the cells were fixed with 4% formaldehyde in PBS for 30 min at 4 °C after three rinses with PBS to preserve their integrity, architecture, and staining. Micrographs were obtained with a confocal laser scanning microscope Zeiss LSM880 by diode laser 405-30.

### Statistical Analysis

Data represent the mean with standard deviation (SD, ±) from three independent experiments, with significance (*p* < 0.05) determined using the ANOVA test in OriginPro software.

## Supporting Information Available

Results of the functionalization of BNC in oxalic acid and choline chloride-based DES at 4 and 6 h (Figure S1 and S2) and micrographs at 20 μm of scale about the differentiation of 3T3-L1 cells in BNC–COOH hydrogel at 3 h (Figure S3) (PDF)
Moreover, the hydrogel of BNC–COOH texture at 3, 4, and 6 h of functionalization by oxalic acid and choline chloride-based DES (Movie S1) (MOV)
A 3D visualization of BNC scaffolds in confocal microscopy (Movie S2) (AVI)
A 3D visualization of BNC–COOH scaffolds in confocal microscopy (Movie S3) (AVI)

## Supplementary Material

am4c22475_si_001.pdf

am4c22475_si_002.mov

am4c22475_si_003.avi

am4c22475_si_004.avi
