# Supplementary material for: Enhanced Cell Proliferation and Maturation Using Carboxylated Bacterial Nanocellulose Scaffolds for 3D Cell Culture
Source: ACS Appl Mater Interfaces. 2025 Mar 5;17(11):16632–43. doi: 10.1021/acsami.4c22475 (PMC11931477; doi:10.1021/acsami.4c22475)
Supplement: Supplementary file 1 — am4c22475_si_001.pdf [file am4c22475_si_001.pdf]

Supporting Information

Enhanced Cell Proliferation and Maturation Using Carboxylated Bacterial Nanocellulose Scaffolds for 3D Cell Culture

Elizabeth Mavil-Guerrero[1], José Manuel Romo-Herrera[2], Priscila Quiñonez-Angulo[1], Francisco J. Flores-Ruiz[3], Edén Morales-Narváez[1], J. Félix Armando Soltero[4], Josué D. Mota-Morales[1]*, Karla Juarez-Moreno[1]*

[1]Centro de Física Aplicada y Tecnología Avanzada (CFATA), Universidad Nacional Autónoma de México (UNAM), Querétaro 76230, México

[2]Centro de Nanociencias y Nanotecnología, Universidad Nacional Autónoma de México, Ensenada, Baja California 22800, México

[3]SECIHTI–Instituto de Física, Benemérita Universidad Autónoma de Puebla, Ciudad Universitaria, Edif. IF-1, Puebla 72570, México

[4]Centro Universitario de Ciencias Exactas e Ingenierías, Universidad de Guadalajara, Guadalajara, Jalisco 44430, México

(*) Author to whom correspondence should be addressed: kjuarez@fata.unam.mx; jmota@fata.unam.mx

Supplementary materials are as follows:

-Supplementary Movie S1: the hydrogel of BNC-COOH texture at 3, 4, and 6 hours of functionalization by oxalic acid and choline chloride-based DES.

- Supplementary Movie S2: Video of the BNC fibers in confocal microscopy. The video is not in real-time.

- Supplementary Movie S3: Video of the BNC-COOH fibers in confocal microscopy. The video is not in real-time.

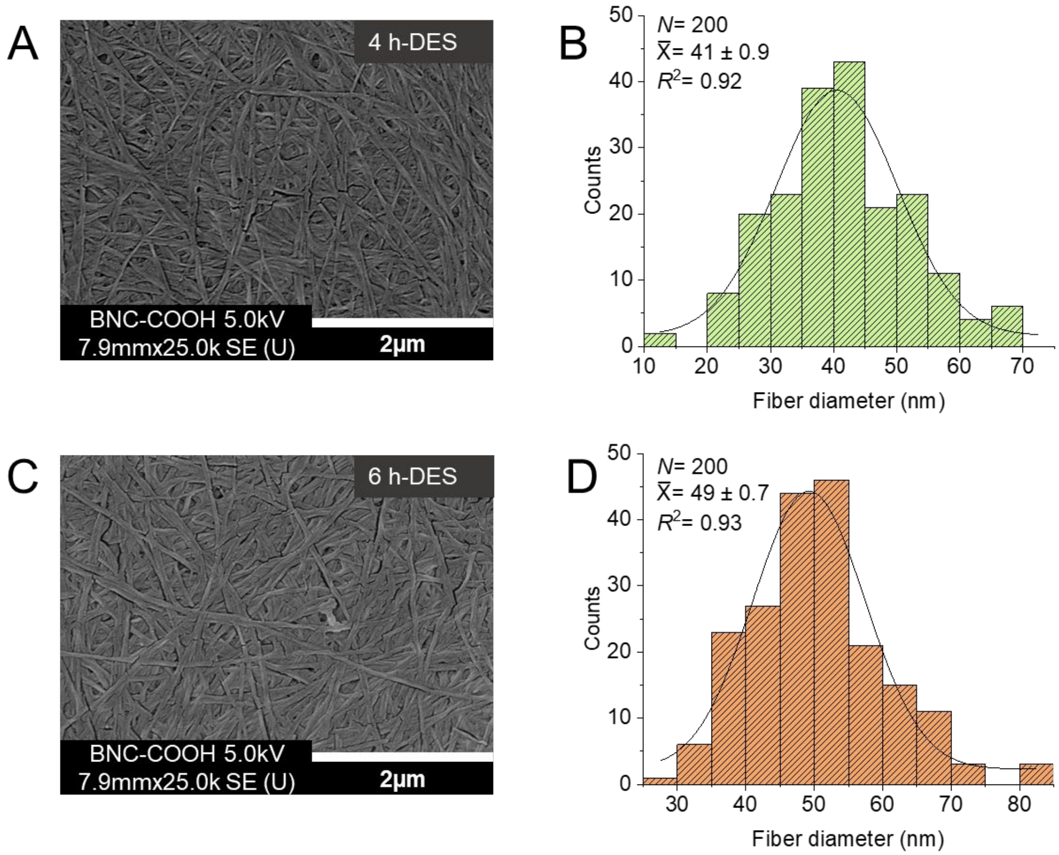

Figure S1. Morphology and size of dried BNC-COOH after 4 and 6 hours of functionalization in a ChCl-OA DES. (A-D) HR-SEM micrographics and corresponding fiber diameter histogram of BNC-COOH 4 h and BNC-COOH 6 h, respectively. Fiber diameter distribution was calculated using ImageJ software. The hydrogels of BNC-COOH were lyophilized and gold-coated by vacuum deposition. Scale bars represent 2 μm.

In Figure S1, the micrographs of BNC-COOH functionalized with ChCl-OA for 4 and 6 hours reveal irregular fibrils with broken and uneven segments. Additionally, the fiber diameter increased to 41 nm and 49 nm for 4 and 6 hours of functionalization, respectively, both exceeding the diameter observed at 3 hours (30 nm) in the dry state.

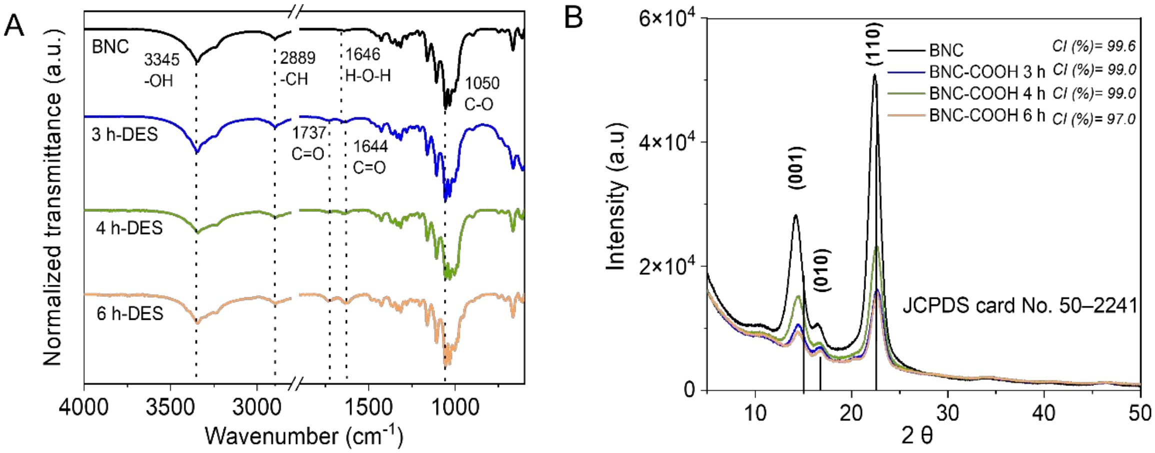

Figure S2. ATR-FTIR spectra and crystallinity of BNC and functionalized BNC-COOH by DES esterification. (A) ATR-FTIR spectra of BNC-COOH by ChCl-OA DES at different times. (B) The crystallinity index (CI) and patterns of BNC-COOH at 3, 4, and 6 hours of functionalization. The hydrogels of BNC-COOH were lyophilized before the characterization.

All FTIR spectra show the typical bands for cellulose, expect the distinct bands due to carboxylic acid functionalization which increased their intensity along the functionalization time (Figure S2 A). On the other hand, the increase in diameter observed by SEM characterization can be attributed to the progressive swelling of the fibers, most likely resulting from a modification of the crystallinity, as evidenced by the overall decrease in the crystallinity index (Figure S2 B).

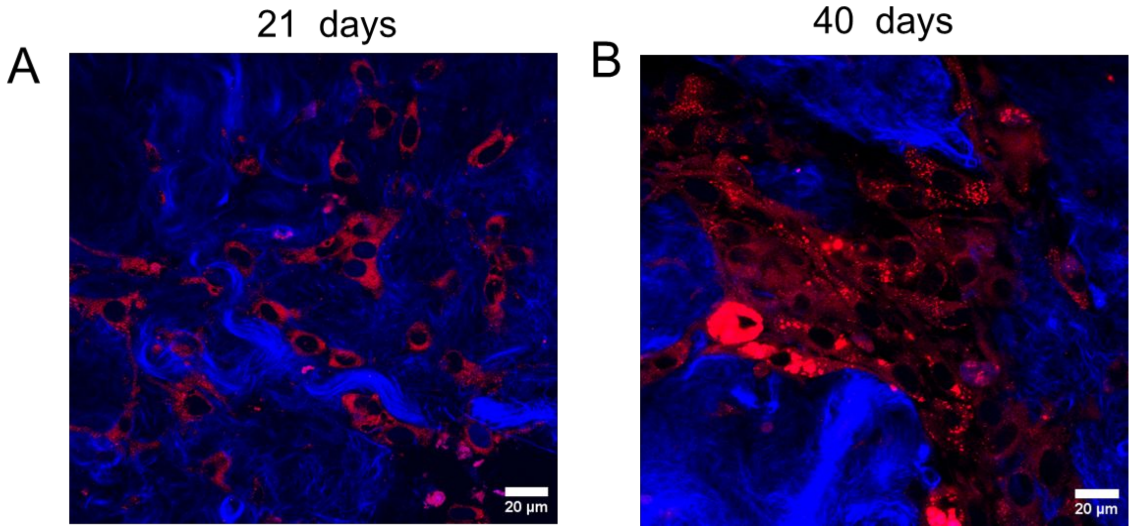

Figure S3. Differentiation of 3T3-L1 cells in BNC-COOH hydrogel. Confocal microscopy images of 3T3-L1 cells (red color) stained with Nile red, along with bacterial nanofibers (blue color) stained with white of calcofluor at (A) 21 and (B) 40 days of culture. Scale bars represent 20 µm. The intense red spots correspond to lipid droplets, which indicate cell maturation.
